# An Analysis of Clinical Characteristics of Rare Bilateral Cerebral Peduncular Infarction

**DOI:** 10.3389/fneur.2019.01107

**Published:** 2019-10-25

**Authors:** Hao Chen, Qian Hu, Hafiz Khuram Raza, Sandeep Singh, Pabitra Rai, Jienan Zhu, Guiyun Cui, Xinchun Ye, Chuanying Xu, Jia Jing, Yonghai Liu

**Affiliations:** ^1^Department of Neurology, The Affiliated Hospital of Xuzhou Medical University, Xuzhou, China; ^2^School of International Education, Xuzhou Medical University, Xuzhou, China; ^3^Department of Biology, Georgia State University, Atlanta, GA, United States

**Keywords:** bilateral cerebral peduncular infarction, “Mickey Mouse ears” sign, locked-in syndrome, persistent vegetative state, neuroimaging

## Abstract

**Objective:** To investigate the anatomical characteristics, clinical manifestations, and imaging features of bilateral cerebral peduncular infarction.

**Methods:** A retrospective analysis was performed on 11 patients diagnosed with bilateral cerebral peduncular infarction in the Affiliated Hospital of Xuzhou Medical University from December 2014 to December 2018. Their clinical and imaging features were analyzed and summarized in combination with the relevant national and international literature.

**Results:** Among all the patients, there were eight cases with a history of hypertension, four cases with a history of diabetes mellitus, and four cases with a history of smoking. Conscious disturbance was observed in nine cases, quadriplegia in seven cases, pseudobulbar paralysis in three cases, and ataxia in one case. Brain magnetic resonance (MR) scans of bilateral cerebral peduncles showed patchy abnormal shadows with a hypointense signal on T1-weighted imaging (T1WI) and apparent diffusion coefficient (ADC) and hyperintense signal on T2-weighted imaging (T2WI), fluid-attenuated inversion recovery (FLAIR), and diffusion-weighted imaging (DWI). Computed tomography angiography (CTA) scans of head and neck showed severe stenosis or occlusion of vertebral artery, basilar artery, or posterior cerebral artery. All the patients received standardized treatment for cerebral infarction. Six patients died while five were left disabled.

**Conclusion:** Bilateral cerebral peduncle infarction may be related to cerebral perfusion insufficiency caused by the stenosis or occlusion of vertebrobasilar artery and its branches. The main clinical manifestations are locked-in syndrome and persistent vegetative state. The specific imaging feature of “Mickey Mouse ear”-like infarction is associated with a poor prognosis.

Bilateral cerebral peduncular infarction (BCPI) is a special type of brainstem infarction, it is rare, and its incidence has not been reported in the literature. The main clinical manifestations are locked-in syndrome or persistent vegetative state, and brain MRI-DWI demonstrates the characteristic “Mickey Mouse Ear” sign. The prognosis is very poor. There are few reports about this particular type of cerebral infarction at home and abroad. In order to improve the understanding of clinicians, this article reviews and analyzes 11 patients' clinical data with BCPI diagnosed in our hospital.

## Information and Methods

### General Information

The data from the past 4 years were collected. There were 11 patients with BCPI who were diagnosed in our hospital and included six males (54.5%) and five females (45.5%). The age of onset was 48–77 years with an average age of 65.8 ± 8.8 years. The past history was significant for hypertension in 8 cases (72.7%), diabetes mellitus in 4 cases (36.4%), and smoking in 4 cases (36.4%). All patients did not receive intravenous thrombolysis and endovascular treatment and were excluded from cardiac embolism by echocardiography and electrocardiogram.

## Methods

The clinical data of 11 patients with BCPI admitted to our hospital in the past 4 years were retrospectively analyzed. The auxiliary results of the patients were summarized, including brain CT, brain MRI, brain and neck CTA, electrocardiogram, and cardiac ultrasound. The therapeutic schedule was summarized and the patients' prognosis were evaluated.

## Results

### Clinical Manifestations

All patients had an acute onset and the time between the onset of symptoms and treatment was in ≤72 h. Before the onset of the disease, all the patients suffered from dizziness, nausea, vomiting, blurred vision, and other precursor symptoms, which aggravated with time. Their clinical features included consciousness disturbance in 9 cases (81.8%); quadriplegia in 7 cases (63.6%); pseudobulbar palsy, such as dysarthria, dysphagia, and pharyngeal reflex, in 3 cases (27.3%); and ataxia in 1 case (9.1%). Babinski's sign was positive on both sides in all the patients. On admission, all the patients were graded according to the National Institutes of Health Stroke Scale (NIHSS) (1) to assess the severity of cerebral infarction. The patients were divided into three grades: normal or mild stroke (0–4 points); moderate stroke (5–15 points) (2 cases, 18.2%); and severe stroke (>15 points) (9 cases, 81.8%) (Table 1).

**Table 1:** Breakdown of the 11 patients with BCPI.

**Case**	**Age/Sex**	**Risk factors**	**Clinical symptoms**	**Location**	**Vascular abnormality**	**NIHSS**	**mRS**	**Outcome**
1	77/F	HTN, DM	Disturbance of consciousness, Pseudobulbar paralysis	BCP, Ce	VA	35	4	Death
2	73/F	DM	Disturbance of consciousness, Tetraplegia	BCP, Oc, Th	MAC, PCA, BA	30	4	Death
3	69/F	None	Disturbance of consciousness	BCP, Ce, Th	MAC, PCA	37	5	Death
4	56/M	HTN, Smoking	Locked-in syndrome	BCP, Ce, Oc	PCA, VA	31	4	Death
5	71/F	None	Disturbance of consciousness, Tetraplegia, Pseudobulbar paralysis	BCP, Oc	VA, BA, PCA	20	3	Disability
6	56/F	HTN	Disturbance of consciousness, Tetraplegia, Pseudobulbar paralysis	BCP, Ce, Th	-	13	3	Disability
7	62/M	HTN	Disturbance of consciousness, Tetraplegia	BCP, Ce, Oc, Th	VA	25	4	Disability
8	68/M	HTN, DM, Smoking	Locked-in syndrome	BCP, Oc	BA	8	3	Disability
9	48/M	HTN, Smoking	Disturbance of consciousness	BCP, Ce, Oc	BA, PCA	27	4	Disability
10	76/M	HTN, DM	Disturbance of consciousness, Tetraplegia	BCP, Oc, Th	BA, VA	35	5	Death
11	68/M	HTN, Smoking	Disturbance of consciousness,Ataxia	BCP, Ce, Oc	BA, PCA	36	4	Death

*HTN, hypertension; DM, diabetes mellitus; BCP, Bilateral cerebral peduncles; Ce, Cerebellum; Oc, Occipital lobe; Th, Thalamus*.

### Imaging Examination

All the patients underwent brain magnetic resonance (MR) scans. There were slightly abnormal shadows present in bilateral cerebral peduncle with hypointensity signals on T1-weighted imaging (T1WI) and apparent diffusion coefficient (ADC) and hyperintensity signals on T2-weighted imaging (T2WI), fluid-attenuated inversion recovery (FLAIR), and diffusion-weighted imaging (DWI). Occipital infarction was observed in 8 cases (72.7%), cerebellar infarction in 7 cases (63.6%), and thalamic infarction in 5 cases (45.5%). Among 11 patients, 10 underwent head and neck computed tomography angiography (CTA) while 1 patient failed to complete the vascular examination because of the severity of disease. There were 6 cases with severe stenosis or occlusion in posterior cerebral artery (PCA) (60%), 6 cases with occlusion or stenosis in basilar artery (BA) (60%), and 5 cases with stenosis or occlusion in vertebral artery (VA) (50%) (Table 1).

### Treatment and Outcome

All the patients were given dual antiplatelet agents, lipid-lowering agents, free-radical elimination therapy (2), mitochondrial function protecting agents (3), etc. Patients with onset <48 h were given argatroban anticoagulation. The risk factors of cerebrovascular diseases, such as blood pressure, blood glucose levels, and lipid levels, were controlled within the time period of the acute window phase, and the patients with a large cerebral infarction area were given mannitol to alleviate brain edema and strengthen the nursing care. Upon discharge, Modified Rankin scale (mRS) score was used to evaluate their prognosis (4), and the patients were divided into two grades: good prognosis (0–2 points) and poor prognosis (3–6 points). All the patients had a poor prognosis. Ninety days after the onset of cerebral infarction, we conducted telephone or face-to-face follow-up for five patients with disabilities (45.5%), and four patients were given five points while one patient was given four points. Six deaths (54.5%) were scored at six points (Table 1).

## Discussion

**Figure 1:**
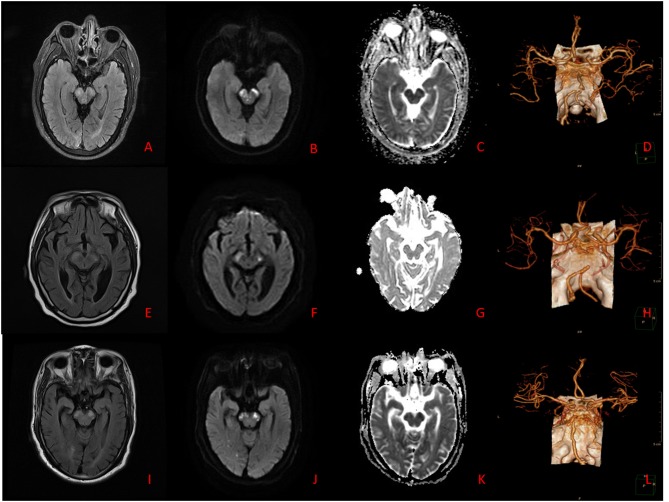
**(A–D)** Case 7. Brain MRI: The lesions appear hyperintense on T2WI, T2-FLAIR, and DWI, and hypointense on ADC. Brain CTA: Stenoses are located in bilateral VA. **(E–H)** Case 8. Brain MRI: The lesions appear hyperintense on T2WI, T2-FLAIR, and DWI, and hypointense on ADC. Brain CTA: Stenosis is located in BA. **(I–L)** Case 10. Brain MRI: The lesions appear hyperintense on T2WI, T2-FLAIR, and DWI, and hypointense on ADC. Brain CTA: Stenoses are located in right VA and BA.

**Figure 2:**
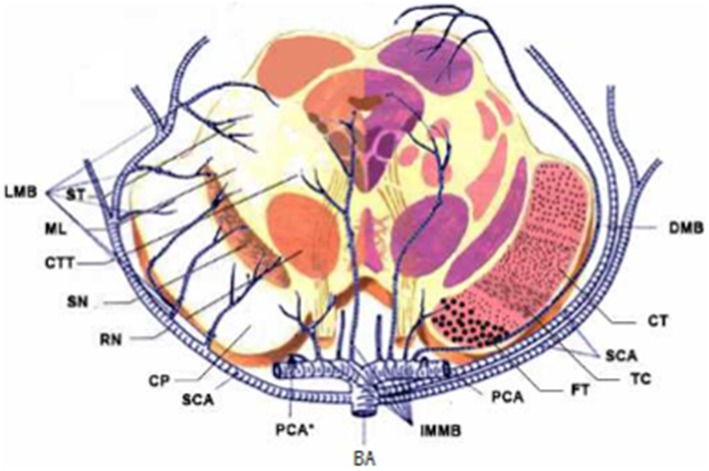
Diagrams of the blood supply of the midbrain. LMB, Lateral Mesencephalic Branches; ST, Spinoreticular Tract; ML, Medial Lemniscus; CTT, Central Tegmental Tract; SN, Substantia Nigra; RN, Red Nucleus; CP, Cerebral Peduncular; SCA, Superior Cerebellar Artery; IMMB, Inferior medial Mesencephalic Branch; FT, Frontopontine Tract; TC, Tractus Corticobulbrais; CT, Corticospinal Tract; DMB, Dorsal Mesencephalic Branch.

**Figure 3:**
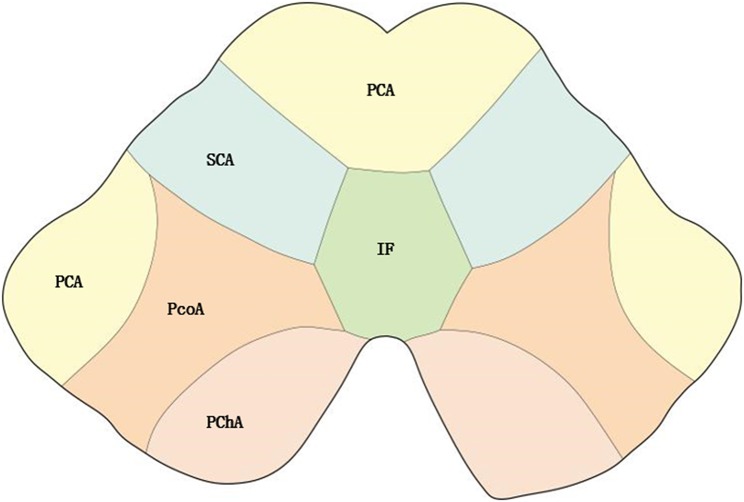
Distribution of blood supply of the midbrain. PChA, Posterior choroidal artery; PcoA, Posterior communicating artery; IF, interpeduncular fossa.

BCPI was first reported by Y. Asakawa et al. and its unique bilateral symmetrical imaging features of cerebral peduncular lesions were called “Mickey Mouse Ear” sign (5). Pure midbrain infarction is rare, accounting for 0.6–2.3% of all ischemic cerebral infarctions, accounting for 8% of all posterior circulation ischemia. Pure bilateral midbrain infarction accounts for 0.12% of all ischemic cerebral infarctions, and BCPI is even more rare, and its prevalence has not been reported. However, it is associated with severe clinical manifestations and a high mortality rate. The clinical characteristics of BCPI are summarized as follows:

According to the distribution of perforating arteries, Goto (6) divided the midbrain into three regions: the middle part, the lateral part, and the posterior part, and the cerebral peduncle consists of the middle and the lateral part, which is supplied by the branches of PCA and SCA (7). PCA can be divided into three groups: the first group is the paramedian branch, which converges with some branches from pontine BA and posterior communicating artery (PcoA); the second group is the short circumflex branch, which converges with branches from SCA and anterior choroidal artery; and the third group is the long circumflex branch, which converges with the quadrilateral artery. The posterior choroidal artery (PChA) is emitted by PCA and is accompanied by a quadrilateral artery that branches along the way to supply the cerebral peduncle. According to the vascular anatomy of the midbrain and the distribution of blood supply areas, the midbrain is supplied by SCA, PcoA, PChA, SCA, and IF (Figures 2, 3), while the cerebral peduncle is mainly supplied by SCA, PcoA, and PChA, and the branches of the midbrain all originate from the vertebrobasilar artery. In this study, 10 patients underwent head and neck CTA scans. Six patients had stenosis or occlusion in PCA, six patients in BA, and five patients in VA. As shown in Figure 1, all three cases had typical vertebrobasilar artery stenosis. Therefore, BCPI is related to deficiency in cerebral perfusion caused by stenosis and occlusion of vertebrobasilar artery and its branches.

BCPI is mainly differentiated from bilateral middle cerebellar peduncle (BMCP). The middle cerebellar peduncle is mainly supplied by the anterior inferior cerebellar artery (AICA) and also receives the blood supply of SCA that is consistent with the AICA terminal branch. So, the middle cerebellar peduncle is located in the watershed area of the above two arteries, and the lack of blood perfusion in the watershed area is considered to be a mechanism of middle cerebellar peduncle infarction (8).

The causes and risk factors of BCPI are the same as those of posterior circulation cerebral infarction, and atherosclerosis is the most common cause of BCPI (9) while hypertension, diabetes mellitus, hyperlipidemia, coronary heart disease, and smoking are other common risk factors. According to TOAST classification (10), all of our patients were classified as having large-artery atherosclerosis (LAA), and the main mechanism of BCPI induced by LAA is hypo-perfusion (11).

BCPI with acute onset has progressive deterioration. The most common presenting symptoms are dizziness, nausea, vomiting, or limb weakness, and the patients then gradually develop locked-in syndrome or persistent vegetative state, that is, quadriplegia and conscious disturbance or long-term coma, or even death. The long-term bedridden survivors are unable to take care of themselves, and BCPI is often accompanied by pulmonary infection, lower extremity arteriovenous embolism, and other serious complications. Because the corticomedullary tract passes through the peduncles of the brain, and bilateral corticomedullary tract damage can cause pseudobulbar palsy, three patients in this group had this symptom. In an extrapyramidal system, occipitotemperopontine tract and frontopontine tract are the connective fibers of cerebral cortex, brainstem, and cerebellum, leading to ataxia after injury (12), but the symptoms of cerebellar ataxia are mild and rarely accompanied by concomitant nystagmus. In this group, one patient had shown unequivocal ataxia as one of the signs and symptoms, while the rest of the patients could not be assessed due to consciousness disorder or quadriplegia. BCPI is a severe cerebral infarction, and most patients present themselves to the hospital with a severe degree of infarction. In this study, NIHSS score was used to evaluate the severity of cerebral infarction, and there were two patients with moderate degree stroke and nine patients with severe degree stroke. NIHSS score is an independent predictor of post-stroke outcome, which is helpful to assess the prognosis of patients with acute stroke (13). The patients with NIHSS score >14 score have higher mortality rate and poor prognosis. BMCP is a frontal ponto-occipital cerebellar fiber pathway, with less severe symptoms than BCPI, chiefly manifested as dizziness, vertigo, paroxysmal or intermittent dysarthria, horizontal nystagmus, and ataxia of limbs and trunk, with or without hearing loss or distortion.

A brain MR scan is more sensitive than CT to posterior circulation infarction, especially brainstem infarction, which is conducive to the early diagnosis and treatment of BCPI. In particular, DWI sequence shows bilateral cerebral peduncle hyperintensity signals, showing the sign of “Mickey Mouse ears,” which is characteristic for its diagnosis (Figure 1). Simple middle cerebral infarction is rare, often accompanied by infarction of adjacent parts. In this group of patients, brain MR scans showed involvement of the occipital lobe in eight cases, cerebellum in seven cases, and thalamus in five cases. BMCP brain MR scans showed bilateral symmetrical pontobrachial infarction, which should be differentiated from BCPI.

Although the infarct area in BCPI is small, its anatomical structure is complex with multiple nerve fibers and conduction pathways, and it is often accompanied by infarctions of adjacent structures, leading to poor prognosis. After admission, all the 11 patients received standardized treatment for cerebral infarction, and those with large area infarction were treated with corticosteroids or mannitol dehydration therapy to control the risk factor. Some patients with severe symptoms were transferred to the intensive care unit while some severe patients chose to abandon the treatment and were discharged. On follow-up, we found that 6 of the 11 patients had died and five were left with disabilities. Their prognosis may be related to the patients' age, general condition, risk factors of cerebrovascular disease, severity of neurological deficit, complications, and mRS scores. Kim et al. (14) reported that the independent predictive prognostic factors of posterior circulation infarction were moderate-to-severe stroke (MTSS) associated with weakness, decreased consciousness, and progressive neurological deficits, in which NIHSS >5 score was defined as acute ischemic stroke. The cases we studied are consistent with the literature report.

## Conclusion

Since the lesions occupy a special anatomical location, BCPI brain MR scans show “Mickey Mouse ears” appearance. This characteristic sign is helpful for clinicians in facilitating the early diagnosis and early treatment of BCPI, which is of great clinical significance for the prognosis.

The limitation of this study is that it was a retrospective study and the records could not be completely pursued in each case. Besides, the number of studies included in this study was low, and there was no comparative study in the control group.

## Data Availability Statement

The datasets generated for this study are available on request to the corresponding author.

## Ethics Statement

This study has been approved by the Ethics Committee of the Affiliated Hospital of Xuzhou Medical University.

## Author Contributions

HC prepared the draft. QH wrote the paper. HR and SS reviewed the paper. PR edited the manuscript. JZ, CX, and JJ helped in data collection. GC and XY analyzed the data. YL supervised the study.

### Conflict of Interest

The authors declare that the research was conducted in the absence of any commercial or financial relationships that could be construed as a potential conflict of interest.
